# Simultaneous Enhancement of Flame Resistance and Antimicrobial Activity in Epoxy Nanocomposites Containing Phosphorus and Silver-Based Additives

**DOI:** 10.3390/molecules28155650

**Published:** 2023-07-26

**Authors:** Tăchiță Vlad-Bubulac, Corneliu Hamciuc, Diana Serbezeanu, Ana-Maria Macsim, Gabriela Lisa, Ion Anghel, Dana-Maria Preda, Yuri Kalvachev, Cristina Mihaela Rîmbu

**Affiliations:** 1 Department of Polycondensation and Thermally Stable Polymers, “Petru Poni” Institute of Macromolecular Chemistry, 41A Grigore Ghica Voda Alley, 700487 Iasi, Romania; chamciuc@icmpp.ro (C.H.); diana.serbezeanu@icmpp.ro (D.S.); macsim.ana@icmpp.ro (A.-M.M.); 2 Department of Chemical Engineering, Faculty of Chemical Engineering and Environmental Protection, “Gheorghe Asachi” Technical University of Iasi, 73 Bd. Mangeron, 700050 Iasi, Romania; gapreot@yahoo.com; 3 Fire Officers Faculty, Police Academy “Alexandru Ioan Cuza”, 3 Morarilor St., Sector 2, 022451 Bucharest, Romania; ion.anghel@academiadepolitie.ro (I.A.); mariadana523@yahoo.com.sg (D.-M.P.); 4 Institute of Catalysis, Bulgarian Academy of Sciences, Acad. G. Bonchev St., bl.11, 1113 Sofia, Bulgaria; kalvachev@ic.bas.bg; 5 Department of Public Health, “Ion Ionescu de la Brad” Iasi University of Life Sciences, 8 Sadoveanu Alley, 707027 Iasi, Romania; crimbu@yahoo.com

**Keywords:** phosphorus-containing bisphenol, zeolite-silver nanoparticles, epoxy nanocomposites, thermal stability, flame resistant, antimicrobial activity

## Abstract

The design and manufacture of innovative multifunctional materials possessing superior characteristics, quality and standards, rigorously required for future development of existing or emerging advanced technologies, is of great importance. These materials should have a very low degree of influence (or none) on the environmental and human health. Adjusting the properties of epoxy resins with organophosphorus compounds and silver-containing additives is key to the simultaneous improvement of the flame-resistant and antimicrobial properties of advanced epoxy-based materials. These environmentally friendly epoxy resin nanocomposites were manufactured using two additives, a reactive phosphorus-containing bisphenol derived from vanillin, namely, (4-(((4-hidroxyphenyl)amino)(6-oxido-6H-dibenzo[c,e][1,2]oxaphosphinin-6-yl)methyl)-2-methoxyphenyl) phenylphosphonate (BPH), designed as both cross-linking agent and a flame-retardant additive for epoxy resin; and additional silver-loaded zeolite L nanoparticles (Ze–Ag NPs) used as a doping additive to impart antimicrobial activity. The effect of BPH and Ze–Ag NPs content on the structural, morphological, thermal, flame resistance and antimicrobial characteristics of thermosetting epoxy nanocomposites was investigated. The structure and morphology of epoxy nanocomposites were investigated via FTIR spectroscopy and scanning electron microscopy (SEM). In general, the nanocomposites had a glassy and homogeneous morphology. The samples showed a single glass transition temperature in the range of 166–194 °C and an initiation decomposition temperature in the range of 332–399 °C. The introduction of Ze–Ag NPs in a concentration of 7–15 wt% provided antimicrobial activity to epoxy thermosets.

## 1. Introduction

Epoxy resins represent one of the most important classes of polymers due to their useful characteristics such as good mechanical, thermal and electrical properties, adhesion to many substrates, chemical and corrosion resistance, optical transparency and so on. They are used in many advanced applications such as adhesives, coatings and paints, as well as in polymer matrices for composites and nanocomposites [1]. Due to their capacity to easily produce complicated geometries quickly and affordably, epoxy resins have recently drawn attention in additive manufacturing, replacing traditional materials and techniques in various fields [2]. The properties of epoxy thermosets can be tuned by appropriately modifying of the structures of epoxy resins, the curing agents and the nature of the cross-linking process, or by incorporating useful additives or fillers [3]. The reinforcement of epoxy resins via the incorporation of appropriate inorganic particles led to improved mechanical properties and can enhance other useful properties (thermal, electric and magnetic characteristics, antimicrobial activity, etc.) in the obtained polymer materials [1,4,5].

Despite many advantages of epoxies, their high flammability and low thermostability at elevated temperatures represent a major disadvantage and limitation [6]. When burning, they produce large amounts of heat and smoke, which makes them unusable in certain industrial fields where flame-resistant materials are required. An efficient approach to improving their flame resistance is to incorporate halogen-containing flame-retardants into epoxy resins. However, the combustion of such systems releases large amounts of toxic and corrosive gases, which are very harmful to the environment and human health; thus, rigorous legislative directives have drastically restricted their widespread use [7]. Consequently, keeping a minimal environmental impact while fireproofing and recycling epoxy thermosets is a crucial desideratum if epoxy resins are to be further considered as highly effective materials designed for cutting-edge technologies [8].

The presence of phosphorus atoms in the structure of the cured epoxy resins, even in low concentration, can improve their flame resistance [9,10,11]. Phosphorus compounds are environmentally friendly and exhibit low toxicity. They can reduce the flammability of polymeric materials by acting both in vapor phase through a radical mechanism to interrupt the combustion process and in condensed phase by facilitating the formation of a layer of carbonaceous residue that acts as a barrier against heat transfer and the diffusion of combustible gases and smoke [9,12,13].

9,10-Dihydro-9-oxa-10-phosphaphenanthrene-10-oxide (DOPO) and its derivatives have been shown to be very effective in reducing the flammability of epoxy resins. DOPO exhibits superior thermal and thermo-oxidative stability, acting in the gas and condensed phases to increase flame resistance. It contains a reactive P-H group that allows for the preparation of a large number of compounds derived from it. Incorporating nitrogen or silicon atoms alongside DOPO groups, in the structure of such derivatives, can create a synergistic effect, leading to remarkable enhancements in thermal stability and a notable reduction in the material’s flammability [13,14,15]. Aromatic phenylphosphonates resulting from phenylphosphonic dichloride and phenols can be incorporated into epoxy resins, improving their flame resistance. They decompose at a relatively low temperature, forming a char layer that prevents combustion [12,16]. Recently, much research has been done to obtain biobased flame retardants [17,18]. For example, vanillin, a biobased compound, has been used as a reactant in the preparation of some compounds used to increase the flame resistance of epoxy resins [19,20].

Intensive research has been undertaken to develop materials with antimicrobial activity that have applications in many areas such as the medical and electronic fields. For example, hospital-acquired infections may occur due to bacterial contamination of the medical equipment and inert surfaces [21]. Therefore, the study and uses of antimicrobial agents have become more frequent in various fields. The most-used method to impart antimicrobial activity to epoxy thermosets is the incorporation of different agents [22,23,24,25,26,27,28]. Among the inorganic antimicrobial agents, silver (Ag) has been extensively used because it has been found to be less toxic compared to other metals, making it suitable for biomedical applications. Even at low concentrations, it shows a wide spectrum of antimicrobial activity [29]. 

Zeolites are microporous crystalline aluminosilicates. Sodium ions present in zeolites can be easily substituted with silver ions to impart to the zeolite antimicrobial activity against bacteria and inhibitory effects toward fungi and viruses [29]. The contact of the bacterial cell with silver zeolite can transfer silver ions to the cell [30]. The diffusion rate of Ag ions out of the zeolite is lower when compared to loading silver ions directly into the polymer matrix; thus, it is possible to increase the silver release time where it is necessary for the materials to show antimicrobial activity for a long period of time. The bacterial cell that makes contact with silver zeolite takes up their silver ions, which inhibit several functions in the cell and consequently cause damage [31]. 

Silver–zeolite can be used as a polymer filler to confer antimicrobial activity to the resulting composites [32]. This compound was incorporated into different polymers such as polyurethane, polyethylene, polysulfone, polyvinyl chloride, poly(vinyl alcohol), silicon elastomers, chitosan, etc., to impart antimicrobial activity [29]. In our laboratory, we prepared silylated Zeolite-L nanoparticles ion exchanged with silver ions and incorporated them into a poly(ether ether ketone) at concentrations of 2, 7 and 12 wt%. The resulting polymer composites exhibited antimicrobial activity and low cytotoxicity [33]. Moreover, recently, electrospun composite membranes based on copoly(ether imide)s generated from Jeffamine and further doped with silver-loaded zeolite L nanoparticles have been successfully produced and characterized [34]. 

The innovative aspect of this study lies in the development of environmentally friendly epoxy resin nanocomposites using two unique additives. The first additive is a reactive phosphorus-containing bisphenol derived from vanillin, specifically (4-(((4-hydroxyphenyl)amino)(6-oxido-6H-dibenzo[c,e][1,2]oxaphosphinin-6-yl)methyl)-2-methoxyphenyl) phenylphosphonate (BPH). This compound serves a dual purpose as both a cross-linking agent and a flame-retardant additive for epoxy resin. The second additive comprises silver-loaded zeolite L nanoparticles (Ze–Ag NPs), which act as doping agents to provide antimicrobial activity to the nanocomposites. The study focuses on examining the influence of BPH and Ze–Ag NPs content on various key characteristics of the thermosetting epoxy nanocomposites, including their structural, morphological, thermal, flame resistance and antimicrobial properties. To investigate the structure and morphology of the epoxy nanocomposites, techniques such as FTIR spectroscopy and scanning electron microscopy (SEM) were employed. The results demonstrated that the nanocomposites exhibited a homogeneous and glassy morphology. The samples displayed a single glass transition temperature ranging from 166 to 194 °C and an initiation decomposition temperature ranging from 332 to 399 °C. One notable finding was that the incorporation of Ze–Ag NPs at concentrations of 7–15 wt% conferred antimicrobial activity to the epoxy thermosets. This discovery highlights the potential of these nanocomposites in inhibiting microbial growth, thereby offering promising applications in antimicrobial materials. Overall, the study presents a novel approach to developing environmentally friendly epoxy resin nanocomposites with enhanced properties, providing insights into their potential applications in various fields.

## 2. Results and Discussion

### 2.1. Synthesis and Characterization of BPH 

The determination of BPH’s structure was accomplished through the use of FTIR and NMR spectroscopy. In the FTIR spectrum, distinct absorption bands were observed at specific wavenumbers: 3070 cm^−1^ (indicating aromatic C–H bonds), 1600 and 1596 cm^−1^ (representing aromatic C=C bonds), 1373 cm^−1^ (corresponding to C–N bonds), 1200 cm^−1^ (associated with P=O groups), 928 cm^−1^ (reflecting P–O–Ar linkages), 753 cm^−1^ (deformation vibration that usually is characteristic for 1,2-disubstituted aromatic DOPO rings) and 815 cm^−1^ (indicative of the deformation of *p*-phenylene rings) (Figure S1).

The structure of BPH was characterized by ^1^H NMR, ^13^C NMR and ^31^P NMR (Figure S2). The appearance of the signals in the NMR spectra is complicated by the phosphorus atom’s presence in the structure. The chirality of the phosphorus stereocenter in the phosphaphenanthrene unit was described as the cause of this phenomenon in the literature [35,36]. In the case of BPH phenylphosphonate, the NMR spectroscopy evidenced the presence of diastereomers a and b in a molar ratio of 1:0.8. The ^1^H NMR spectrum of BPH displays some characteristic signals: the signals corresponding to protons from the methoxy group appeared in the proton spectrum as a doublet in the interval 3.53–3.56 ppm; OH group displayed broader resonance (8.53–8.56 ppm), while the proton of the CH group gave two multiplets (for both isomers) at 4.95 ppm and 5.39 ppm, respectively; the aromatic protons gave complicated signals in the region of 6.88–8.21 ppm, while the proton of NH was bound to the multiplets located at 5.73 ppm and 6.14 ppm, respectively.

In the ^13^C NMR spectrum, the signals corresponding to the carbon atom of the methoxy group appeared as a singlet at 55.6 ppm. The CH group exhibits multiplets at 56 ppm and 57 ppm, whereas the aromatic carbon atoms display signals ranging from 119 ppm to 150 ppm. The quaternary carbon directly bonded to oxygen appeared most deshielded within the range of 148.8–149.86 ppm.

The presence of doublet signals in the ^31^P spectrum provides evidence for the existence of two isomers. This is particularly evident in DOPO, where two distinct signals are observed at 28.7 and 31.5 ppm for the two phosphorus atoms. However, when it comes to the P-linked phenyl group, the signals for the two isomers overlap, resulting in a single signal at 12.5 ppm.

In order to understand the influence of the BPH flame-retardant additive on the envisaged resin formulations in terms of flame retardancy and thermal stability, thermogravimetry analysis has been performed. According to Figure S2d, the monomer undergoes a multi-step decomposition process characterized by various mechanisms. These mechanisms include random chain cleavage resulting in smaller fragments, breaking of end bonds, elimination of side units, cross-linking, diffusion, vaporization, initiation cycles involving recombination and gas phase reactions. Nevertheless, the compound revealed moderate thermal stability starting to decompose at temperatures above 250 °C as a consequence of the cleavage of P–O–C bonds, which are well known as more sensitive to degradation at elevated temperatures [37,38]. The decomposition rate experienced a decline within the temperature range of 550–700 °C, indicating the emergence of a more heat-resistant residue. The presence of a substantial amount of char residue provides further support for this hypothesis.

### 2.2. Structural and Morphological Characterization of Epoxy-Based Nanocomposites

**Figure 1:**
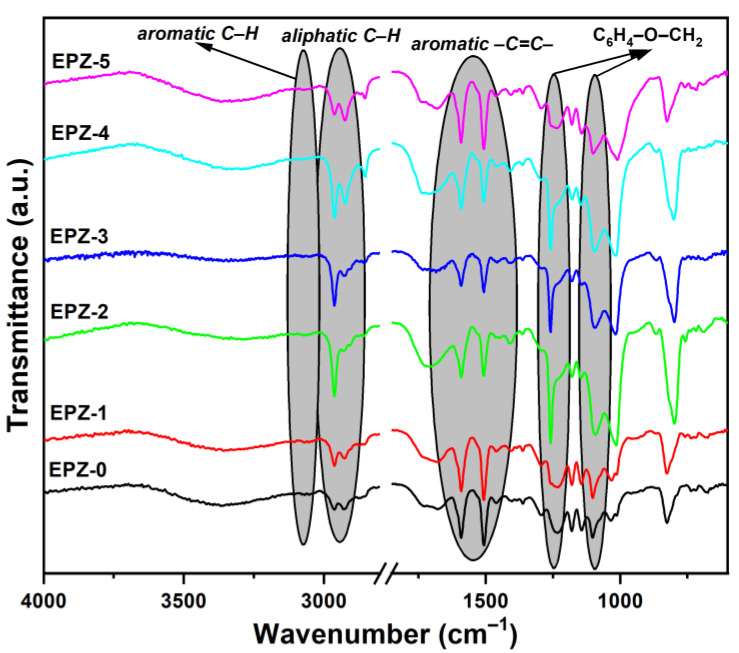
FTIR spectra of epoxy nanocomposites.

The structures of the resulting thermosets were investigated via FTIR spectroscopy (Figure 1). The FTIR spectrum of neat EP-0 showed characteristic absorption bands at 3368 (N–H and O–H groups), 3060 (aromatic C–H), 2962, 2925, 2874 (aliphatic C–H), 1590 and 1507 (aromatic –C=C–) and 1235 and 1032 (C_6_H_4_–O–CH_2_ asymmetric and symmetric stretching vibration, respectively).

**Figure 2:**
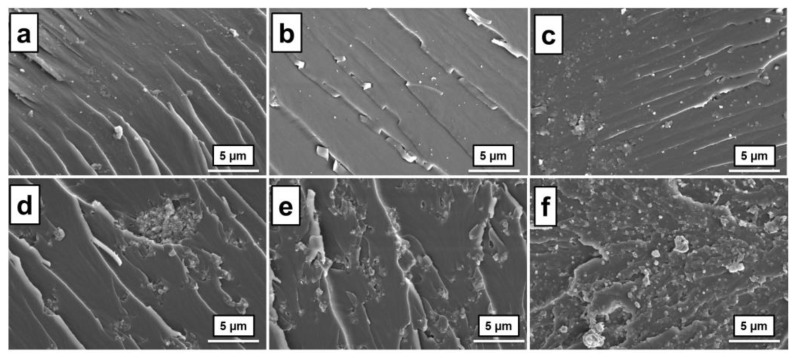
SEM micrographs of epoxy nanocomposites: EPZ-0 (**a**); EPZ-1 (**b**); EPZ-2 (**c**); EPZ-3 (**d**); EPZ-4 (**e**); EPZ-5 (**f**).

Figure 2 illustrates the SEM micrographs of the fracture surfaces of the samples. EPZ-0, EPZ-1, EPZ-2 and EPZ-4 showed a smooth surface with a glassy and homogeneous structure. In the case of EPZ-3 and EPZ-4, it can be seen that the zeolite nanoparticles were distributed on the fracture surface. The fracture surface of the sample containing a higher content of Ze–Ag nanoparticles (EPZ-5, 15 wt% Ze–Ag) showed higher surface roughness, and nanoparticle agglomerations were observed.

In Figures S3 and S4, we present the EDX diagram for EPZ-2 and EPZ-3, where the presence of P, C, O, N and S atoms on the fracture surface of EPZ-2 was evidenced, while along all these elements, additional elements, such as Si, Na, Ag and Al coming from Ze–Ag additive, could be observed in the case of EPZ-3, thus confirming the uniform dispersion of the zeolite additive in the mass of EPZ-0 formulation. 

### 2.3. Thermal Characterization of EPZ Nanocomposites

**Figure 3:**
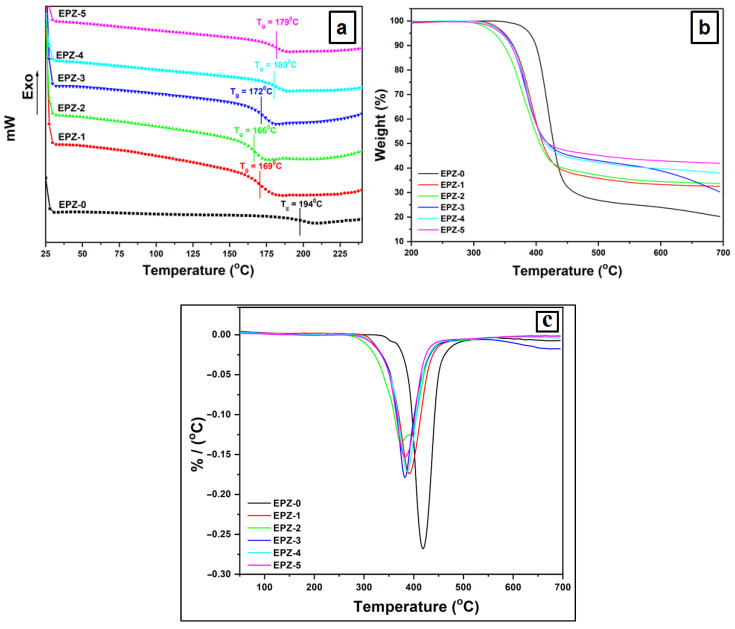
DSC curves (**a**) TGA (**b**) and DTG curves (**c**) of epoxy resin nanocomposites.

The glass transition temperature (*T_g_* midpoint) was determined by differential scanning calorimetry measurements (DSC). The DSC curves showed a single *T_g_*, suggesting the existence of a homogeneous system (Figure 3a). The *T_g_* values of the samples were in the temperature range of 166–194 °C (Table 1). As expected, EPZ-0 showed the highest *T_g_* value (194 °C), which can be explained by the presence of a higher crosslinking density as a result of using as the crosslinking agent only DDS diamine, and the absence of other inorganic additives. The use of bisphenol BPH as a cross-linking agent together with DDS led to a decrease in *T_g_* for EPZ-1 and EPZ-2. This behavior can be due to the higher molecular weight of the BPH and the occurrence of steric effects as a result of the DOPO bulky group presence. Moreover, the presence of some unreacted oxirane groups may be responsible for the decreased *T_g_* as a consequence of the lower reactivity of phenolic groups of BPH in comparison to amino groups of DDS. Compared to the EPZ-1, the samples that contain Ze–Ag nanoparticles showed slightly higher *T_g_* values, suggesting the existence of some interactions between the inorganic nanoparticles and the macromolecular chains of the polymers.

The thermal stability of the nanocomposites was investigated using dynamic thermogravimetric analysis (TGA). Some of the parameters determined by this method from the thermogravimetric curves (Figure 3) are shown in Table 1. The samples showed an initial decomposition temperature (*T*_5_) in the range of 322–399 °C (Figure 3b). As expected, the introduction of the bisphenol BPH containing two DOPO groups and a main chain phenylphosphonic unit led to a significant reduction of *T*_5_. Thus, EPZ-0 had a *T*_5_ value of 399 °C, while those for EPZ-1 and EPZ-2 were 349 and 332 °C, respectively. The samples containing Ze–Ag nanoparticles showed *T*_5_ values close to that of EPZ-1.

The 30 wt% weight loss temperatures (*T*_30_) were in the ranges of 315–389 °C. The sample EPZ-0 showed the lowest value of *T*_30_ (315 °C), while the other samples containing phosphorus exhibited higher values, in the range of 379–389 °C, thus highlighting the effect of the compound with phosphorus in increasing the amount of residue as a result of the material decomposition at high temperatures.

The heat resistance index (*T_HRI_*) was determined by using the following relation [39]:

*T_HRI_* = 0.49 [*T*_5_ + 0.6(*T*_30_ − *T*_5_)].

*T_HRI_* quantifies the resistance to a heat flow of a polymer composite. An increase of *T_HRI_* was observed by introducing phosphorus-containing BPH and Ze–Ag nanoparticles, suggesting slightly better thermal resistance compared to the neat EPZ-0.

The decomposition residue at 700 °C was in the range of 19.8–41.8 wt%. A significant increase in its value was observed by introducing BPH as a cross-linking agent. Thus, if the reference sample had a residue value of 19.8 wt%, EPZ-1, which contains 1 wt% phosphorus, had a residue value of 32.5 wt%. The highest value of its residue exhibited—as expected—EPZ-5 containing 1 wt% phosphorus and 15 wt% Ze–Ag nanoparticles (41.8 wt%).

The temperature at which the weight loss rate was maximum (*T_max_*) was in the range of 368–412 °C, as was determined from the DTG curves (Figure 3c). 

A significant decrease in *T_max_* was observed for EPZ-1 and EPZ-2, where both DDS and BPH were introduced as crosslinking agents. Moreover, it is observed that the introduction of BPH led to a significant decrease in the maximum rate of decomposition of the material, suggesting that lower quantities of combustible gases are released per unit time. This behavior correlates very well with the *HRR* values as a function of temperature (MCC Analysis), where a significant decrease in *PHHR* is observed for samples containing phosphorus.

**Figure 4:**
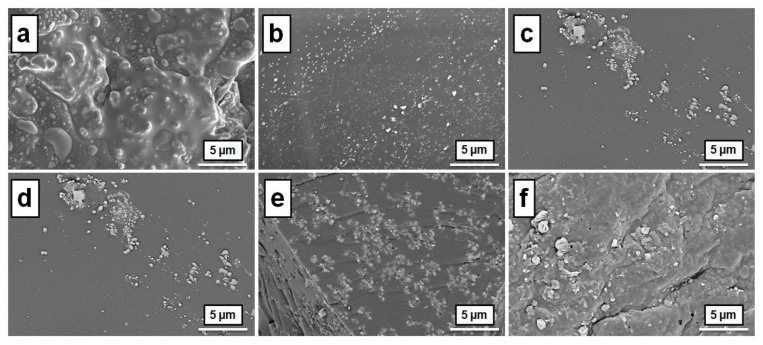
SEM micrographs of the char of the epoxy resin nanocomposites after pyrolysis: EPZ-0 (**a**), EPZ-1 (**b**), EPZ-2 (**c**), EPZ-3 (**d**), EPZ-4 (**e**) and EPZ-5 (**f**).

Figure 4 presents SEM micrographs of the char which resulted from heating the samples up to 700 °C with the heating rate of 10 °C/min, under nitrogen atmosphere. 

As it can be seen the chars of EPZ-1 and EPZ-2 were compact and dense, suggesting that they exhibited good protection in reducing the flammability of epoxy nanocomposites. In the case of the samples containing both phosphorus and Ze–Ag nanoparticles, the chars were dense and compact; however, some holes can be observed distributed on their surface. 

In Figure S5, we present the EDX diagram and EDX mapping of the EPZ-2 residue where the presence of P, C, O, N and S atoms was evidenced. In this case, a homogeneous distribution of P and S atoms was observed. In Figure S6, we present the EDX diagram and EDX mapping of EPZ-3 residue. The presence of elements coming from Ze–Ag (Si, Na, Ag, Al) can be seen. Some agglomerations of Ze–Ag nanoparticles were observed on the surface of the char residue of the EPZ-3, confirming again the presence of the Ze–Ag NPs in the epoxy resin nanocomposites.

**Figure 5:**
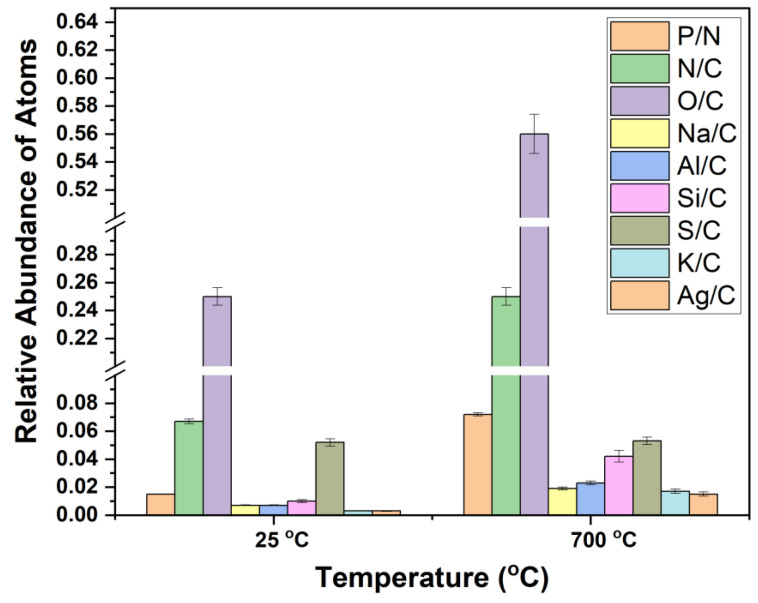
The abundance of P, N, O, Na, Al, Si, S, K and Ag atoms depending on the C atoms in EPZ-3 at room temperature and in the char residues of EPZ-3 at 700 °C.

Furthermore, the semi-quantitative EDX analysis was applied to compare the relative abundance of atoms as a function of temperature. Thus, certain information regarding the mechanism of flame retardancy can be obtained by introspecting the composition of the carbonaceous residue resulting from the thermal degradation of a material subjected to pyrolysis [6,40]. A remarkable increase in the P/C ratio was observed in the case of the carbonaceous residue of EPZ-3 sample compared to the P/C ratio determined for the EPZ-3 sample at room temperature (Figure 5).

Increasing the rate of char deposition in the solid phase—which typically acts as an inhibitor of smoke production and as an anticatalyst of other products evolution in the burning sites—is another way to alter the flammability of finite materials in addition to controlling the release rate of combustible gases during combustion. Numerous studies revealed that phosphorus-containing polymers in general, and in particular compounds including phosphorus atom in phosphaphenanthrene aromatic heterocycles, are responsible for production of enriched and compact layers of char upon thermal decomposition, thus decreasing the exothermicity of the pyrolysis reactions of EPZ and inhibiting the thermal conductivity of the burning materials [41,42]. 

### 2.4. Microscale Combustion Calorimetry (MCC) Tests

**Figure 6:**
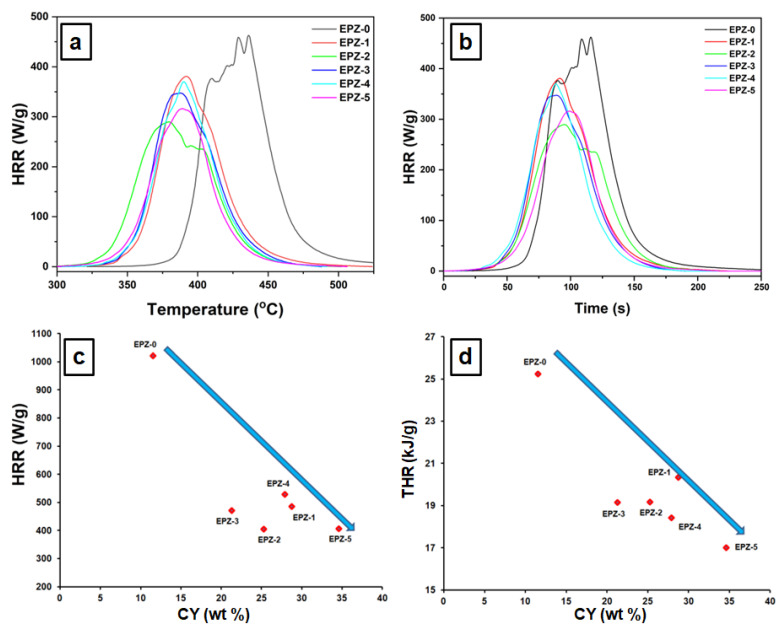
Fire behavior of epoxy resin nanocomposites revealed from heat release rates versus temperature (**a**), heat release rates versus time (**b**), heat release rates versus char yield (**c**) and total heat release versus char yield curves (**d**) for epoxy resin nanocomposites.

The epoxy nanocomposites were subjected to MCC test, the most important data being listed in Table 2. In Figure 6a,b, the HRR curves of the nanocomposites were plotted depending on temperature and time, respectively.

Analyzing Figure 6a, EPZ-0 showed four peaks at different PHRR values, and EPZ-2 presented three peaks. The rest of the samples—EPZ-1, EPZ-3, EPZ-4 and EPZ-5—showed a single peak. The samples containing phosphorus exhibited lower values of PHRR and T_PHRR_ when compared with those of EPZ-0. The lowest values were obtained for EPZ-2 containing 2 wt% phosphorus. Analyzing the HRR curves as a function of time (Figure 6b), it is observed that the first peak of each sample appears around the average value of 92.3 s.

In the micro-level analysis of material fire performance using MCC, the HRC (Heat Release Capacity) is a crucial parameter. It can be determined through MCC tests and serves as a means to classify material flammability. Lower HRC values in MCC tests indicate reduced flammability and a lower risk of fire in real-scale scenarios. Additionally, the THR (Total Heat Release) can serve as an indicator of the overall amount of fuel generated during combustion [43]. The THR is an important characteristic, since low THR implies strong resistance of the samples during the combustion process. An obvious reduction in HRC and THR was observed for the nanocomposites EPZ 1–5 compared to the control sample EPZ-0; thus, the HRC value of EPZ-0 was 1022.59 (J/(g·K)), while for EPZ-1 and EPZ-2, the values were 486.4(J/(g·K) and 405.6 (J/(g·K)), respectively. Moreover, the samples containing Ze–Ag nanoparticles exhibited low values of HRC (407.93 (J/(g·K)) for EPZ-5, 472.08 (J/(g·K)) for EPZ-3 and 530.42 (J/(g·K)) for EPZ-4). THR value for EPZ-0 was 25.25 (kJ/g). A substantial decrease in THR can be noticed for the samples containing phosphorus. The lowest value of THR was observed for EPZ-5 (17.01 kJ/g).

Since the percentage of char (CY) is an indication of the amount of unburned fuel in a material, it is expected that the material with a higher percentage of char will produce less heat during combustion [44]. The amount of residue left after burning each sample was recorded and the percentage mass ratio (CY) between the mass of the residue and the initial mass of the sample was calculated. CY values ranged from 11.50 wt% for EPZ-0 to 34.59% for EPZ-5. It is also observed that the temperature value for the initiation of the decomposition processes for the EPZ-0 control sample was higher compared to the other samples, a fact also observed in the MCC curves. 

It can be stated that in terms of HRC, CY and THR (Figure 6c,d), the nanocomposites EPZ-1–5 showed improvements over the control sample EPZ-0 in terms of fire behavior. Moreover, from the point of view of THR, CY and HRC, EPZ-5 stands out, in which case a decrease in THR of 32.6%, an increase in CY of 66.7% and a decrease in HRC of 60.3% compared to the control sample appeared. 

The thermal resistance of the materials is highlighted by a low HRC and THR and a high Char Yield. According to the results presented comparatively in Figure 6c,d, the best results were obtained for the sample EPZ-5 sample.

### 2.5. Antimicrobial Activity

Techniques for testing antimicrobial activity are based on the release of antimicrobial molecules and their diffusion into a solid or liquid medium containing a given microbial load. Depending on the structure and properties of the matrices to be tested, microbiological methods are used and adapted to determine antimicrobial activity.

**Figure 7:**
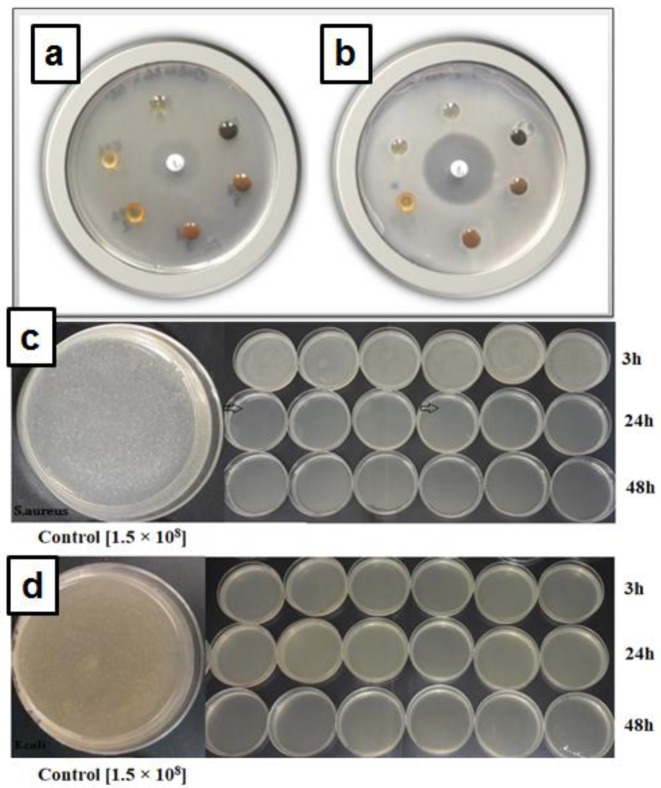
Kirby–Bauer diffusion method testing of antimicrobial activity of epoxy resin pellets embedded with zeolite-L and silver nanoparticles against *S. aureus* (**a**) and *E. coli* (**b**), and contact time method testing against *S. aureus* (**c**) and *E. coli* (**d**).

The tests using the diffusion technique showed that the antimicrobial substances contained in the epoxy resin did not diffuse into the structure of the culture medium and interacted with the microbial cells only upon direct contact; thus, they lacked the inhibition zones that indicate antimicrobial activity against *Staphylococcus aureus* and *Escherichia coli* using this technique (Figure 7a,b).

Silver-containing nanocomposites are known for their antimicrobial activity against a large variety of microorganisms due to their size, shape, charge, high surface-to-mass ratio, and high reactivity [25,45,46]. Considering these properties, the nanocomposites based on epoxy resin and nanoparticles of zeolite-L and silver were also tested via the contact time technique. The results were surprising and showed very good activity against both *Staphylococcus aureus* (Gram-positive bacteria) and, in particular, *Escherichia coli* (Gram-negative bacteria) (Table 3).

The multiplication of *Staphylococcus aureus* species after 3 h of incubation in the presence of epoxy nanocomposite pellets was exuberant, not quantifiable, and similar to the control (1.5 × 10^8^ cfu/mL). After 24 h, the log reduction in all samples analyzed varied from 5.6588 (EPZ-3) to 6.318 (EPZ-5), with a reduction percentage > of 99.9999%. After 48 h of contact, the epoxy resin nanocomposites—EPZ-1, EPZ-2, EPZ-4 and EPZ-5—completely inhibited the suspension of *Staphyococcus aureus* (100%) (Table 3, Figure 7c), with the exception of samples EPZ-0 and EPZ-3, where a lower but relevant inhibitory effect (99.9999%) was observed. The behavior of epoxy resin nanocomposites against *Escherchia coli* was different from that observed with *Staphylococcus aureus*. All types of epoxy pellets with zeolite and silver (EPZ-0, EPZ-1, EPZ-2, EPZ-4 and EPZ-5) inhibited the microbial suspension after 3 h of incubation, and the effect was maintained up to 24/48 h of contact in the liquid environment. (Table 3, Figure 7d).

Studies show that silver nanoparticles exert different antimicrobial effects against bacterial species depending on the structure of the bacterial wall, although it is known that Gram-positive bacteria (*Staphylococcus aureus*) have a higher sensitivity to most antimicrobial agents [33,47,48]. However, in our study, the combination of zeolite and silver embedded in epoxy resins produced a higher inhibitory effect on the Gram-negative species (*Escherichia coli*) than on the Gram-positive species (*Staphylococcus aureus*).

Similar studies conducted with nanocomposites based on epoxy resins showed the cumulative antimicrobial effect depending on the presence of clay and reported a microbial percentage of 87.9% against *Staphylococcus aureus* and 94.9% against *Escherichia coli* [49]. A relatively recent study [50] demonstrated the antimicrobial mechanism of composite materials based on clay and metal ions (iron, aluminum, etc.), which, by simultaneous action, damage lipopolysaccharides on the outer membranes of *Escherichia coli* species, leading to oxidative stress and the release of free radicals capable of damaging DNA and bacterial proteins, resulting in cell death. 

For the same reasons, and considering the recognized antimicrobial activity of silver nanoparticles, we can hypothetically deduce that their association with zeolite-L and the inclusion in epoxy resins act through the same intimate mechanisms that have led to the inhibition of more than 99% of *Staphylococcus aureus* and *Escherichia species.*

## 3. Materials and Methods

### 3.1. Materials

The DOPO compound, specifically 9,10-Dihydro-9-oxa-10-phosphaphenanthrene-10-oxide, was acquired from Chemos GmbH in Altdorf, Germany. Prior to its utilization, it underwent a fresh dehydration. Vanilin, phenylphosphonic dichloride, 4-aminophenol, *N*-methyl-2-pyrrolidone (NMP), triethylamine (Et_3_N), 4,4′-diaminodiphenylsulfone (DDS) and AgNO_3_ were provided by Sigma-Aldrich (St. Louis, MO, USA) and used without further purification. The bisphenol A diglycidyl ether-based epoxy resin, having the epoxy equivalent weight (EEW) of 0.53 equiv. per 100 g (Mn ~ 377 g/mol), was acquired from Sigma-Aldrich. Bis(4-formyl-2-metoxyphenyl) phenylphosphonate was prepared via a condensation reaction of vanillin with phenylphosphonic dichloride, as described in the literature [51].

### 3.2. Synthesis of bis(4-(((4-Hidroxyphenyl)amino)(6-oxido-6H-dibenzo[c,e][1,2]oxaphosphinin-6-yl)methyl)-2-methoxyphenyl) Phenylphosphonate (BPH)

**Scheme 1:**
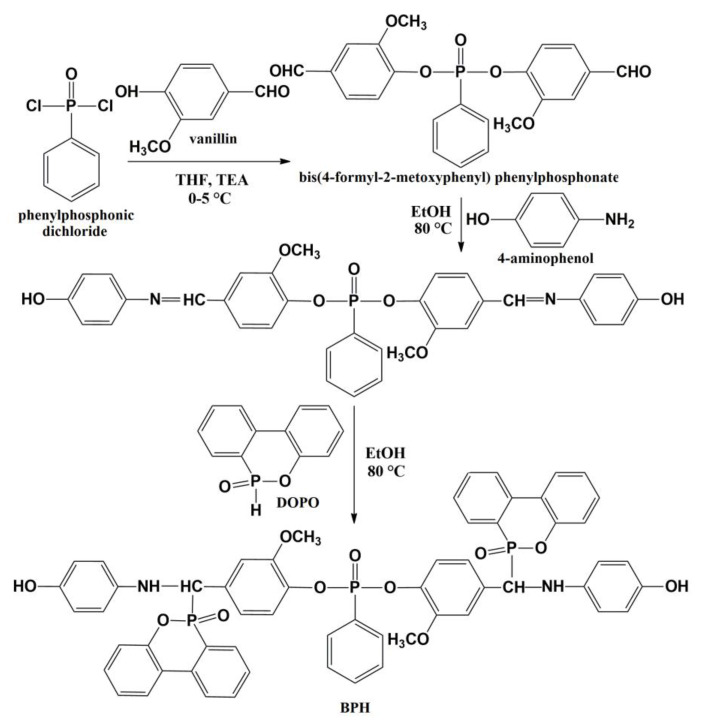
Preparation of BPH.

BPH was prepared using an adapted method, previously described [52] (Scheme 1). The synthesis of bisphenol was carried out in one pot in two stages: in the first stage, the reaction between bis(4-formyl-2-metoxyphenyl) phenylphosphonate and 4-aminophenol took place, resulting in a bisphenol containing two imine groups; in the second stage, DOPO was added and underwent an additional reaction at the imine groups. 

Yield = 85%.

FTIR (KBr, cm^−1^): 3070, 1600, 1596, 1373, 1200, 928, 815, 753.

^1^H NMR (600.13 MHz, DMSO-*d*_6_, ppm): 3.53–3.56 (7.3H, dd, J = 6.3 Hz, 6.1 Hz, OCH_3_), 4.95–5.39 (1.8H, m, CH both isomers), 5.72–6.13 (1.8H, m, NH both isomers), 6.41–6.58 (7.3H, dd, J_H-P_ = 9.1 Hz, 9.1 Hz, H14, H15, H17, H18 both isomers), 6.88–7.02 (4.7H, m, H2 isomer a, H24, H21), 7.09–7.13 (2H, m, H20), 7.18–7.22 (1H, m, H2 isomer b), 7.29 (1.9H, t, J = 6.91 Hz, H4), 7.42 (3H, t, J = 7.9Hz, H3), 7.57–7.61 (2.8H, m, H27, H10), 7.70–7.74 (2.2H, m, H28), 7.78 (1.2H, t, J = 7.6 Hz, H9), 7.89–7.92 (1.7H, m, H26), 8.5 (1H, m, H11), 8.12–8.21 (3.5H, m, H5, H8), 8.53–8.56 (1.7H, s, OH).
^13^C (100.61 MHz, DMSO-*d*_6_, ppm): 55.58 (s, OCH_3_), 55.8–56.8 (m, CH), 113.5 (s, C20), 115.3 (s, C14, C15, C17, C18), 119.8–120.1 (m, C2), 120.6 (C24, C21), 121.5 (dd, J_C-P_ = 9.8 Hz, J_C-P_ = 10.4 Hz, C6), 122.5 (d, J_C-P_ = 47.6 Hz, C7), 123.4–123.9 (m, C8), 124.6 (d, J_C-P_ = 11 Hz, C4), 125.6 (d, J_C-P_ = 25.2 Hz, C5), 128.1–128.6 (m, C3a (isomer a), C27, C10), 130.3–130.4 (m, C3b (isomer b)), 130.6 (d, J_C-P_ = 16.4 Hz, C28), 131.8 (d, J_C-P_ = 10.6 Hz, C26, C11), 133.2–133.7 (m, C9, C25), 135.2–135.4 (m, C12), 138.2–138.3 (m, C19), 13.0–139.4 (m, C13), 148.7–148.9 (m, C1, C22), 149.4 (s, C16), 149.9 (C23).

^13^P NMR (242.94 MHz, DMSO-*d*_6_, ppm): 28.7–31.5 P in DOPO units, 12.5 P in phenylposhonate units.

Working under hydrothermal conditions, nanoparticles of zeolite L were synthesized with a Si/Al ratio of 4. The average size of these nanoparticles was approximately 200 nm. To silylate zeolite L, it was treated with 3-aminopropyl(diethoxy)-methylsilane. Ze–Ag was then prepared by subjecting 1.5 g silylated zeolite L to a 0.1 N aqueous solution of AgNO_3_ (30 mL) at 70 °C for 6 h, while keeping it away from light. Zeolite L and Ze–Ag were prepared following the previously published procedure [34]. 

### 3.3. Preparation of Epoxy Resin Nanocomposites

The curing process of epoxy resin involved the use of combinations of BPH and DDS as hardeners. The specific compositions of the pre-curing mixtures, which included epoxy resin, BPH, DDS and Ze–Ag, can be found Table 4. BPH was dispersed in the epoxy resin under vigorous stirring at 80 °C for two hours. DDS was added and the mixture was heated at 130 °C to obtain a homogeneous translucent system. The as-prepared mixture was poured into aluminum molds. It was then subjected to thermal curing at different temperatures: 120 °C for 10 h, followed by 1 additional hour each at 140, 160, 180 and 200 °C. To prevent cracking, the resulting thermosets were gradually cooled to room temperature. In the case of samples EPZ-3, EPZ-4 and EPZ-5, prior to mixing, the epoxy resin and Ze–Ag nanoparticles underwent ultrasonication for 30 min at 50 °C. 

### 3.4. Measurements

#### 3.4.1. FTIR Analysis

FTIR spectroscopy was employed to examine the structure of epoxy thermosets. For this purpose, a BioRad “FTS 135” FTIR spectrometer with a Specac “Golden Gate” ATR accessory was utilized. Scans were recorded using a LUMOS Microscope Fourier Transform Infrared (FTIR) spectrophotometer from Bruker Optik GmbH, Ettlingen, Germany. The scans were conducted between 4000 and 600 cm^−1^ at a resolution of 4 cm^−1^, and an attenuated total reflection (ATR) device was incorporated in the setup.

#### 3.4.2. NMR Analysis

The ^1^H, ^13^C and ^31^P NMR spectra were acquired using a Bruker Avance Neo NMR spectrometer operating at different frequencies: 400.13 MHz and 600.13 MHz for ^1^H NMR; 100.61 MHz for ^13^C NMR; and 242.94 MHz for ^31^P NMR, respectively. The chemical shifts (δ) for ^1^H and ^13^C NMR were calibrated with respect to residual solvent peaks (DMSO-*d*_6_) at 2.512 ppm and 39.4761 ppm, respectively.

#### 3.4.3. Scanning Electron Microscopy (SEM)

The epoxy thermosets and their corresponding chars were examined using an Environmental Scanning Electron Microscope (ESEM) known as Quanta 200. The microscope operated at 10 kV with secondary electrons in low vacuum mode, utilizing the LFD detector. Additionally, the Quanta 200 microscope was equipped with an Energy Dispersive X-ray (EDX) system for both qualitative and quantitative analysis, as well as elemental mapping.

#### 3.4.4. TGA Measurements

The thermal stability characteristics (TG and DTG) of BPH and epoxy thermosets were obtained using a Mettler Toledo TGA-SDTA851e equipment, Columbus, OH, USA. The measurements were conducted under a nitrogen atmosphere with a heating rate of 10 °C per minute in the temperature range of 25 °C to 700 °C. The mass of the samples used in the thermogravimetric analysis was between 2.2 and 4.7 mg.

#### 3.4.5. Differential Scanning Calorimetry (DSC) Measurements

The BPH and epoxy thermosets were subjected to differential scanning calorimetry (DSC) analysis using a Mettler Toledo DSC1 instrument. The measurements were conducted in a controlled environment with an inert atmosphere, employing a heating rate of 10 °C per minute. Nitrogen gas was purged at a flow rate of 100 mL per minute, and aluminum crucibles were utilized to hold samples weighing between 2.6 and 5.5 mg.

TG, DTG and DSC curves were interpreted to determine the main characteristics using STAR^e^ software, version 9.0 from Mettler Toledo.

#### 3.4.6. Microscale Combustion Calorimetry (MCC)

Samples were subjected to controlled temperature conditions to assess their flammability using microscale combustion calorimetry (MCC) experiments. The experiments followed the guidelines outlined in “Method A” as specified in ASTM D7309-13 [53].

### 3.5. Antimicrobial Activity

The antimicrobial activity of 6 epoxy resin nanocomposites with zeolite L nanoparticles containing silver ions (Zn–Ag) was tested using two techniques to evaluate their antimicrobial potential. In the first phase, the samples were tested using the modified and adapted Kirby–Bauer diffusion method [54]. The epoxy nanocomposites loaded with Ze–Ag nanoparticles were modelled as a disk with a diameter of 5 mm. The antimicrobial activity was tested against *Staphylococcus aureus* ATCC 25923 (Gram-positive bacteria) and *Escherichia coli* ATCC 25922 (Gram-negative bacteria).

The bacterial cultures that had been incubated for 24 h were used to prepare cell suspensions. These suspensions were adjusted to a density equivalent to a turbidity of 0.5 on the McFarland standard, which corresponds to a concentration of 1.5 × 10^8^ colony-forming units (cfu) per milliliter. Sterile Petri plates containing sterile Mueller–Hinton agar (Oxoid) were prepared by melting the agar and allowing it to cool to 45 °C. Then, 1 mL of bacterial suspension was added to each plate. Ze–Ag epoxy nanocomposite disks (EPZ-0, EPZ-1, EPZ-2, EPZ-3, EPZ-4, EPZ-5) were spread on the surface of the medium.

A standardized gentamicin disc (Oxoid, 10 µg) was used as a positive control. Plates were incubated in a microbiological thermostat (Binder BD23) at 37 °C for 24 h.

Standardized bacterial strains—Staphylococcus aureus ATCC 25923 and Escherichia coli ATCC 25922—were purchased from Thermo Fisher Scientific, Waltham, MA, USA (Microbiology) Romania under catalog numbers R4607010 and R4607050, respectively, and their quality certificates can be found at the address listed here: https://www.thermofisher.com/order/catalog/product/R4607010?SID=srch-srp-R4607010 (accessed on 17 July 2023).

The genetic Information on the tested strains can be found on the platform of the cell line supplier American Type Culture Collection (https://www.atcc.org, accessed on 17 July 2023) These bacterial strains are part of the collection of bacterial strains from the Faculty of Veterinary Medicine of the Iasi University of Life Sciences, Iasi, Romania.

**Scheme 2:**
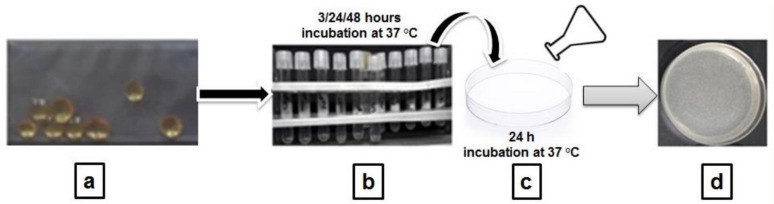
Pathway of antimicrobial activity testing by contact time method (3 h, 24 h, 48 h). EPZ-0, EPZ-1, EPZ-2, EPZ-3, EPZ-4 and EPZ-5 pellet samples (**a**), bacterial suspensions in which the nanocomposite pellets were immersed (**b**), bacterial suspensions distributed into nutrient agar Mueller Hinton (Oxoid) (**c**) and the microbial density developed in the structure of the culture medium (**d**).

To evaluate the antimicrobial activity of the epoxy resin nanocomposites, a quantitative method was also used to evaluate the percentage logarithmic reduction in the number of microbial cells at certain predetermined times (3, 24, 48 h). The same microbial suspensions (1.5 × 10^8^ bacterial cells/mL) of *Staphylococcus aureus* ATCC 25923 and *Escherichia coli* ATCC 25922 were used for this purpose. A pellet of each EPZ-0, EPZ-1, EPZ-2, EPZ-3, EPZ-4 and EPZ-5 sample (a) was mixed with 5 mL of bacterial suspension (b) and vortexed at 2000 rpm for 1 min (Vortex Biosan V-1) (Scheme 2).

After 3 h, 24 h and 48 h incubation at 37 °C (b), from the bacterial suspensions in which the nanocomposite pellets were immersed, 1 mL was taken and distributed into Petri plates over which was added the nutrient agar Mueller Hinton (Oxoid), which was melted and cooled to 45 °C (c). After solidification, the plates were incubated (37 °C) for 24 h to determine the microbial density developed in the structure of the culture medium (d) and quantified in the form of colony forming units (cfu/mL).

## 4. Conclusions

The primary aim of this study was to develop innovative epoxy-based thermosetting composites suitable for additive manufacturing. This was achieved by adjusting the thermal properties, flame retardancy and antimicrobial properties of the epoxy resin through the use of multiple material components and synergistic additives. To enhance flame retardancy, a phosphorus-containing bisphenol (BPH) was employed as a co-curing agent. Additionally, Ze–Ag nanoparticles were utilized to introduce antimicrobial activity in the composites. The results of thermogravimetric analysis revealed that the inclusion of BPH, along with phosphorus and Ze–Ag nanoparticles, led to an increased residue amount at 700 °C, indicating improved thermal stability and a dense, hole-free structure. The MCC test demonstrated enhanced flame resistance and reduced heat release capacity for samples containing phosphorus or both phosphorus and Ze–Ag nanoparticles. Furthermore, after 48 h of contact, the epoxy resin nanocomposites—EPZ-1, EPZ-2, EPZ-4 and EPZ-5—completely inhibited the suspension of S. aureus. Similarly, all types of epoxy pellets containing zeolite and silver (EPZ-0, EPZ-1, EPZ-2, EPZ-4 and EPZ-5) suppressed the microbiological suspension of *E. coli* after 3 h of incubation, with the effect persisting for 24–48 h in a liquid environment.

## Figures and Tables

**Table 1:** Thermal properties of epoxy resin nanocomposites.

Sample	*T_g_*^1^ (°C)	*T*_5_^2^ (°C)	*T*_10_^3^ (°C)	*T*_30_^4^ (°C)	*T_HRI_* (°C)	*T_max_*^5^ (°C)	Char Yield at 700 °C (wt%)
EPZ-0	194	399	399	315	171	419	19.8
EPZ-1	169	349	369	389	173	391	32.5
EPZ-2	166	332	347	379	176	375	34.1
EPZ-3	172	348	368	385	181	382	30.2
EPZ-4	180	347	367	387	182	387	37.7
EPZ-5	179	345	365	386	181	381	41.8

^1^ Glass transition temperature; ^2^ Temperature at 5 wt% wight loss; ^3^ Temperature at 10 wt% wight loss; ^4^ Temperature at 30 wt% wight loss; ^5^ Temperature at which the mass loss is the highest.

**Table 2:** Data obtained by MCC analysis for the epoxy thermosets.

Samples vs. Data	EPZ-0	EPZ-1	EPZ-2	EPZ-3	EPZ-4	EPZ-5
CY (wt%)	11.5	28.71	25.24	21.26	27.87	34.59
THR (kJ/g)	25.25	20.35	19.18	19.15	18.42	17.01
PHRR 1 (W/g)	376.28	380.90	289.58	347.39	370.03	316.14
T_PHRR_ 1 (^o^C)	410.56	391.97	379.89	387.93	390.13	389.15
Time 1 (s)	90.5	91.5	95.5	89.0	88.5	99.0
PHRR 2 (W/g)	402.32	-	241.91	-	-	-
T_PHRR_ 2 (°C)	421.22	-	395.19	-	-	-
Time 2 (s)	101	-	111	-	-	-
PHRR 3 (W/g)	458.64	-	235.52	-	-	-
T_PHRR_ 3 (°C)	429.25	-	403.08	-	-	-
Time 3 (s)	109	-	119	-	-	-
PHRR 4 (W/g)	462.90	-	-	-	-	-
T_PHRR_ 4 (°C)	436.24	-	-	-	-	-
Time 4 (s)	116	-	-	-	-	-
HRC (J/(g·K))	1022.59	486.74	405.62	472.08	530.42	407.93

CY = char yield; PHRR = heat release peak; THR = total heat release; T_PHRR_ **=** temperature of heat release peak; Time = the time to attain heat release peak; HRC = heat release capacity.

**Table 3:** The antimicrobial activity of epoxy resin nanocomposites pellets.

Species Bacteria	Incubation Time	EPZ-0 ufc/0.5 mL	EPZ-1 ufc/0.5 mL	EPZ-2 ufc/0.5 mL	EPZ-3 ufc/0.5 mL	EPZ-4 ufc/0.5 mL	EPZ-5 ufc/0.5 mL
*S. aureus*	T0 (Control)	1.5 × 10^8^	1.5 × 10^8^	1.5 × 10^8^	1.5 × 10^8^	1.5 × 10^8^	1.5 × 10^8^
	3 h	UQ	UQ	UQ	UQ	UQ	UQ
	24 h	23.4 × 10^1^	23.1 × 10^1^	16.2 × 10^1^	32.9 × 10^1^	19.6 × 10^1^	72 × 10^0^
	Log reduction	5.8068	5.81247	5.9665	5.6588	5.8838	6.318
	% reduction	99.9998	99.9998	99.9998	99.9997	99.9998	99.9999
	48 h	32 × 10^0^	0	0	10.1 × 10^1^	0	0
	Log reduction	6.6709	0	0	6.1717	0	0
	% reduction	99.99997	100	100	99.99993	100	100
*E. coli*	T0 (Control)	1.5 × 10^8^	1.5 × 10^8^	1.5 × 10^8^	1.5 × 10^8^	1.5 × 10^8^	1.5 × 10^8^
	3 h	0	0	0	0	0	0
	24 h	0	0	0	0	0	0
	48 h	0	0	0	0	0	0
	Log reduction	>6	>6	>6	>6	>6	>6
	% reduction	100	100	100	100	100	100

UQ = unquantifiable.

**Table 4:** Preparation of epoxy resin nanocomposites.

Sample	Epoxy Resin (g)	DDS (g)	BPH (g/wt%)	Zeolite-Ag (g/%)	P (%)
EPZ-0	5	1.64	-	-	0
EPZ-1	5	1.55	0.755/10.35	-	1
EPZ-2	5	1.472	1.438/18.179	-	2
EPZ-3	5	1.537	0.868/10.55	0.822/10	1
EPZ-4	4	1.132	0.677/10.65	0.44/7	1
EPZ-5	1	0.2976	0.1759/10.17	0.2559/15	1

## Data Availability

The data that support the findings of the current study are listed within the article.

## Supplementary Materials

The following supporting information can be downloaded at: https://www.mdpi.com/article/10.3390/molecules28155650/s1, Figure S1. FTIR spectrum of BPH. Figure S2. ^1^H NMR (a), ^13^C NMR (b), ^31^P NMR (c) and TG/DTG curves of BPH (d). Figure S3. EDX mapping of EPZ-2. Figure S4. EDX mapping of EPZ-3. Figure S5. EDX mapping of EPZ-2 residue. Figure S6. EDX mapping of EPZ-3 residue.
