# Supplementary material for: Simultaneous Enhancement of Flame Resistance and Antimicrobial Activity in Epoxy Nanocomposites Containing Phosphorus and Silver-Based Additives
Source: Molecules. 2023 Jul 26;28(15):5650. doi: 10.3390/molecules28155650 (PMC10419371; doi:10.3390/molecules28155650)
Supplement: Supplementary file 1 [file molecules-28-05650-s001.zip › molecules-2519965-supplementary.pdf]

Supporting Information

Simultaneous Enhancement of Flame Resistance and Antimicrobial Activity in Epoxy Nanocomposites Containing Phosphorus and Silver-Based Additives

Tăchiță Vlad-Bubulac [1,*], Corneliu Hamciuc [1], Diana Serbezeanu [1], Ana-Maria Macsim [1], Gabriela Lisa [2], Ion Anghel [3], Dana-Maria Preda [3], Yuri Kalvachev [4] and Cristina Mihaela Rîmbu [5]

1 Department of Polycondensation and Thermally Stable Polymers, "Petru Poni" Institute of Macromolecular Chemistry, 41A Grigore Ghica Voda Alley, 700487 Iasi, Romania; chamciuc@icmpp.ro (C.H.); diana.serbezeanu@icmpp.ro (D.S.); macsim.ana@icmpp.ro (A.-M.M.)
2 Department of Chemical Engineering, Faculty of Chemical Engineering and Environmental Protection, "Gheorghe Asachi" Technical University of Iasi, 73 Bd. Mangeron, 700050 Iasi, Romania; gapreot@yahoo.com
3 Fire Officers Faculty, Police Academy "Alexandru Ioan Cuza", 3 Morarilor St., Sector 2, 022451 Bucharest, Romania; ion.anghel@academiadepolitie.ro (I.A.); mariadana523@yahoo.com.sg (D.-M.P.)
4 Institute of Catalysis, Bulgarian Academy of Sciences, Acad. G. Bonchev St., bl.11, 1113 Sofia, Bulgaria; kalvachev@ic.bas.bg
5 Department of Public Health, Iasi University of Life Sciences, 8 Sadoveanu Alley, 707027 Iasi, Romania; crimbu@yahoo.com
* Correspondence: tvladb@icmpp.ro

Figure S1. FTIR spectrum of BPH.

Figure S2. ^{1}H NMR (a), ^{13}C NMR (b), ^{31}P NMR (c) and TG/DTG curves of BPH (d).

Figure S3. EDX mapping of EPZ-2.

Figure S4. EDX mapping of EPZ-3.

Figure S5. EDX mapping of EPZ-2 residue.

Figure S6. EDX mapping of EPZ-3 residue.

Characterization of BPH:

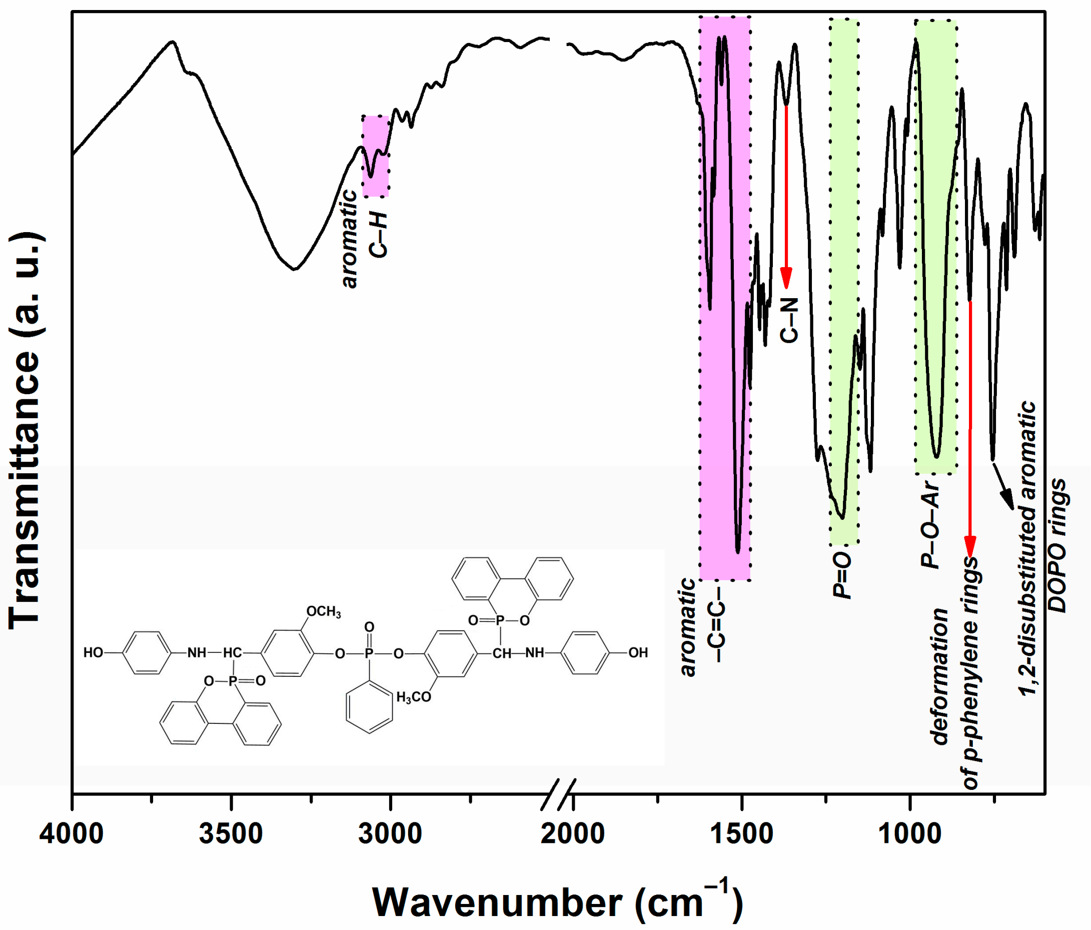

Figure S1. FTIR spectrum of BPH.

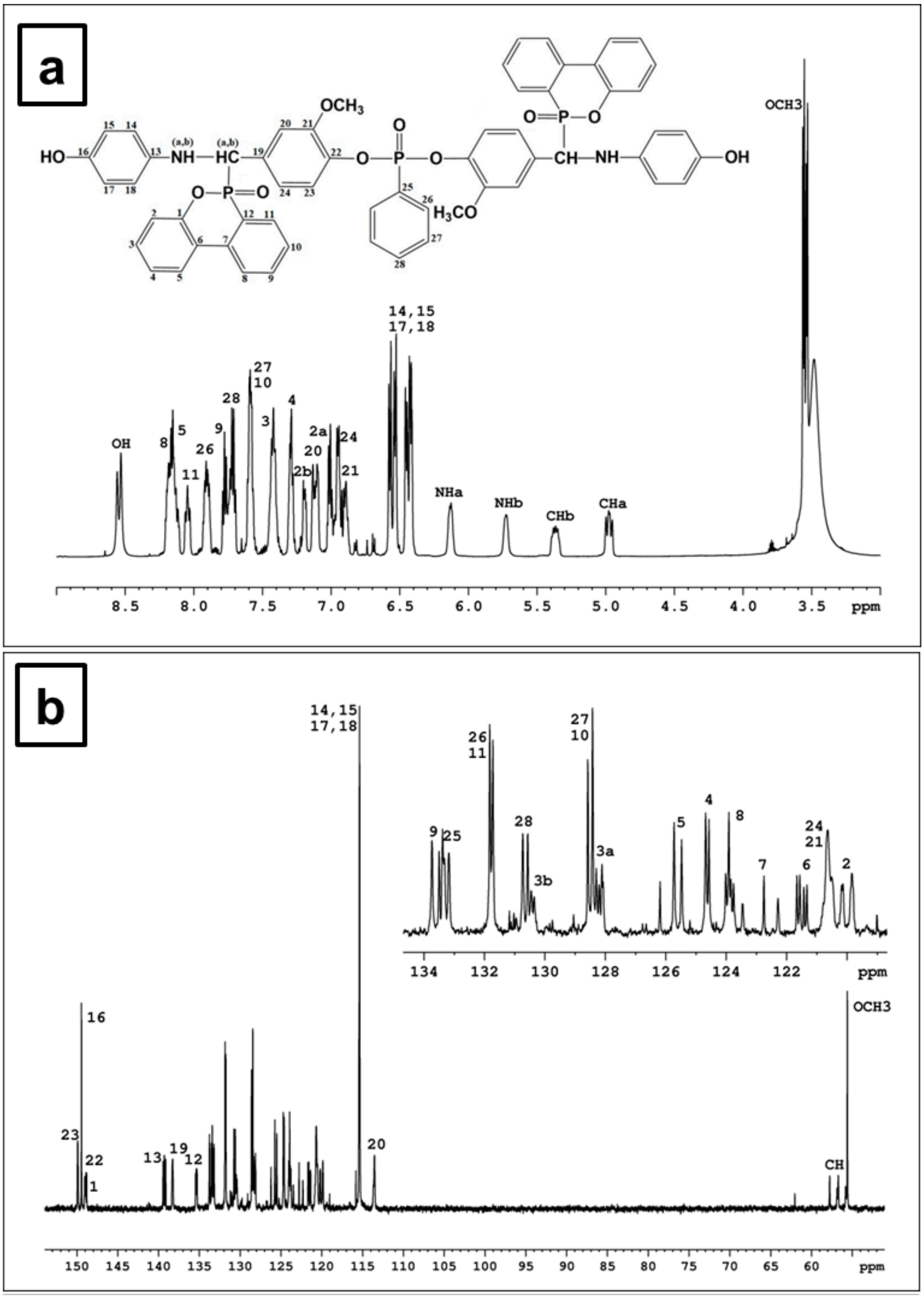
a
OCH3
HO
NH
HC
CH
OH
H3CO
O
P
OH
OCH3
OH
NHa
NHb
CHb
CHa
14,15
17,18
27
10
28
ppm
b
14,15
17,18
26
11
27
10
16
23
22
1
13
19
12
20
CH
OCH3
3a
3b
24
21
ppm

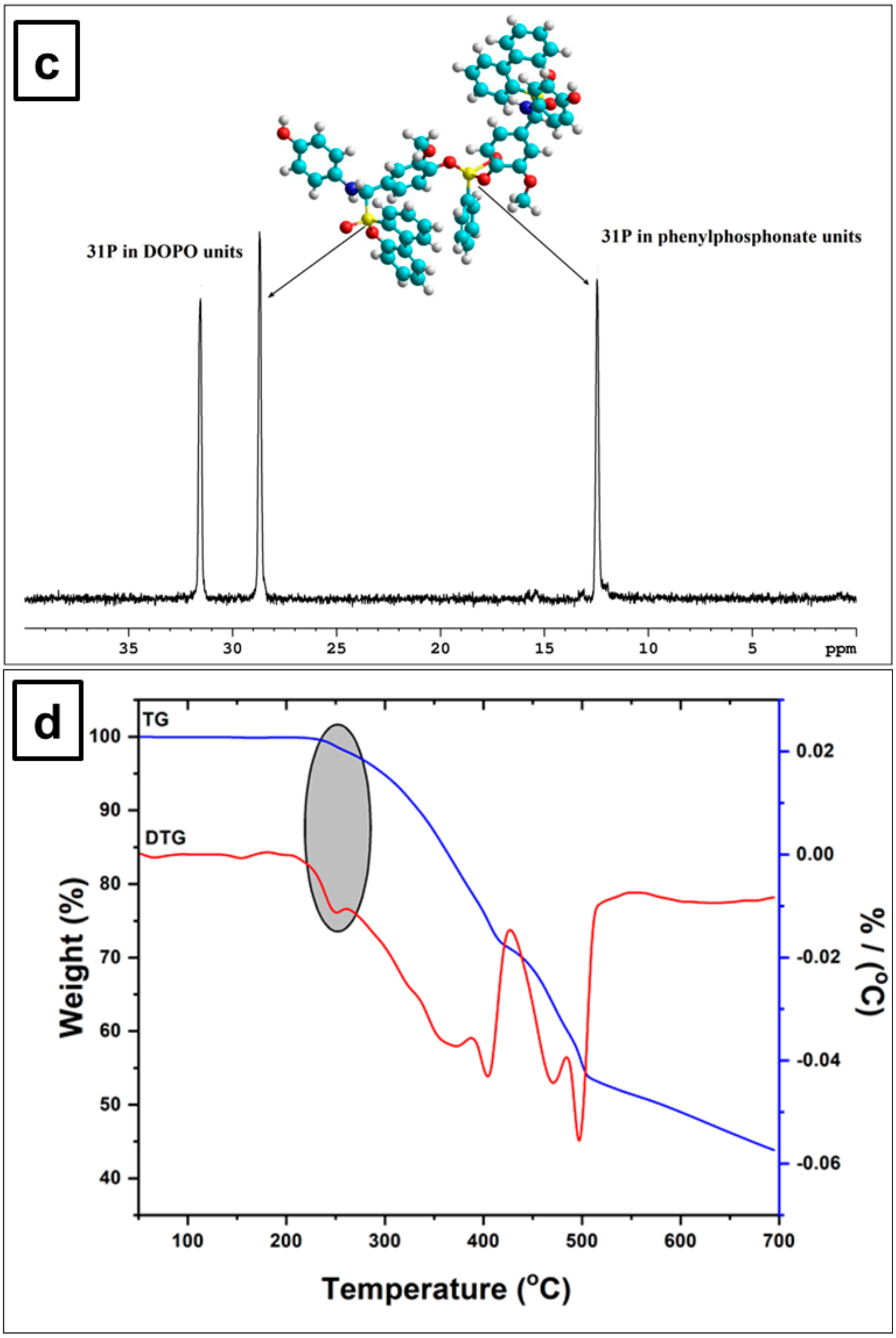

Figure S2. ^{1}H NMR (a), ^{13}C NMR (b), ^{31}P NMR (c) and TG/DTG curves of BPH (d).

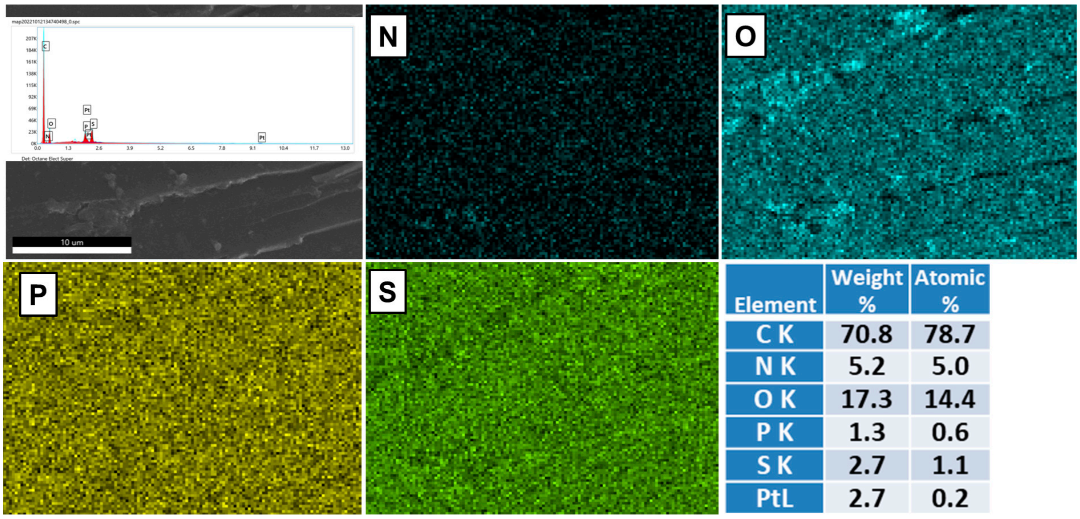

Element	Weight %	Atomic %
C K	70.8	78.7
N K	5.2	5.0
O K	17.3	14.4
P K	1.3	0.6
S K	2.7	1.1
PtL	2.7	0.2

Figure S3. EDX mapping of EPZ-2.

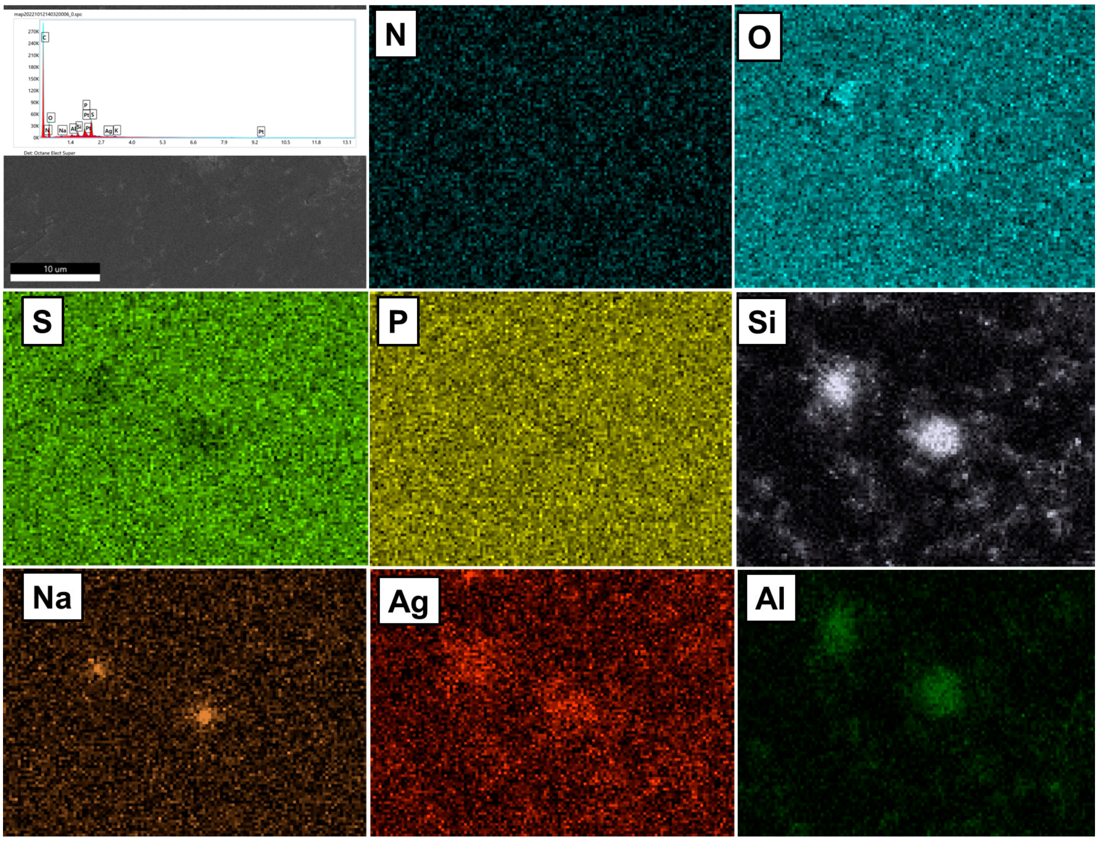

Figure S4. EDX mapping of EPZ-3.

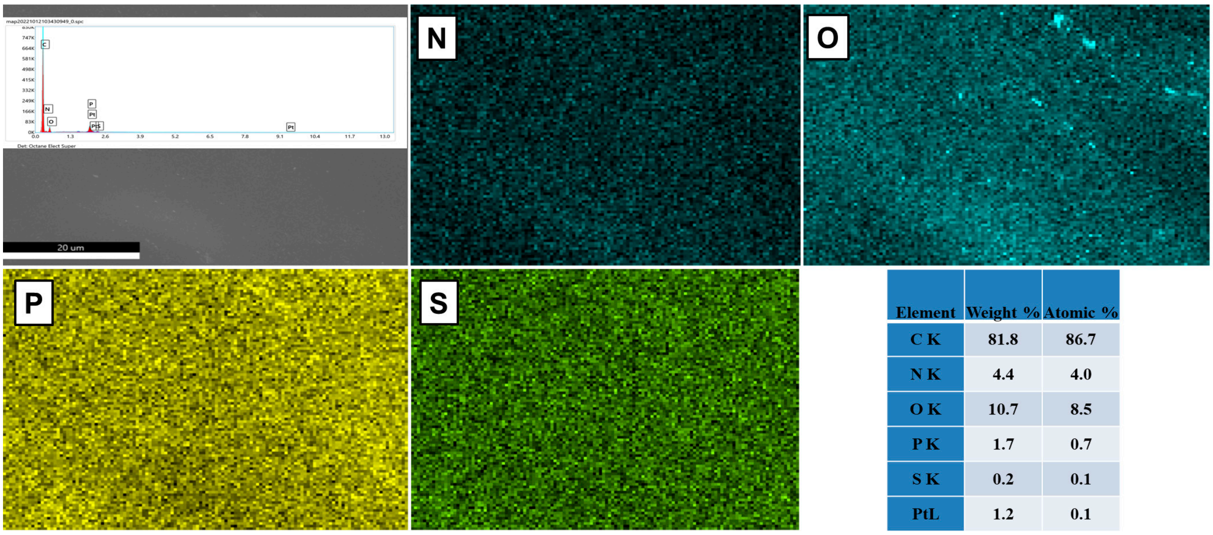

Element	Weight %	Atomic %
C K	81.8	86.7
N K	4.4	4.0
O K	10.7	8.5
P K	1.7	0.7
S K	0.2	0.1
PtL	1.2	0.1

Figure S5. EDX mapping of EPZ-2 residue.

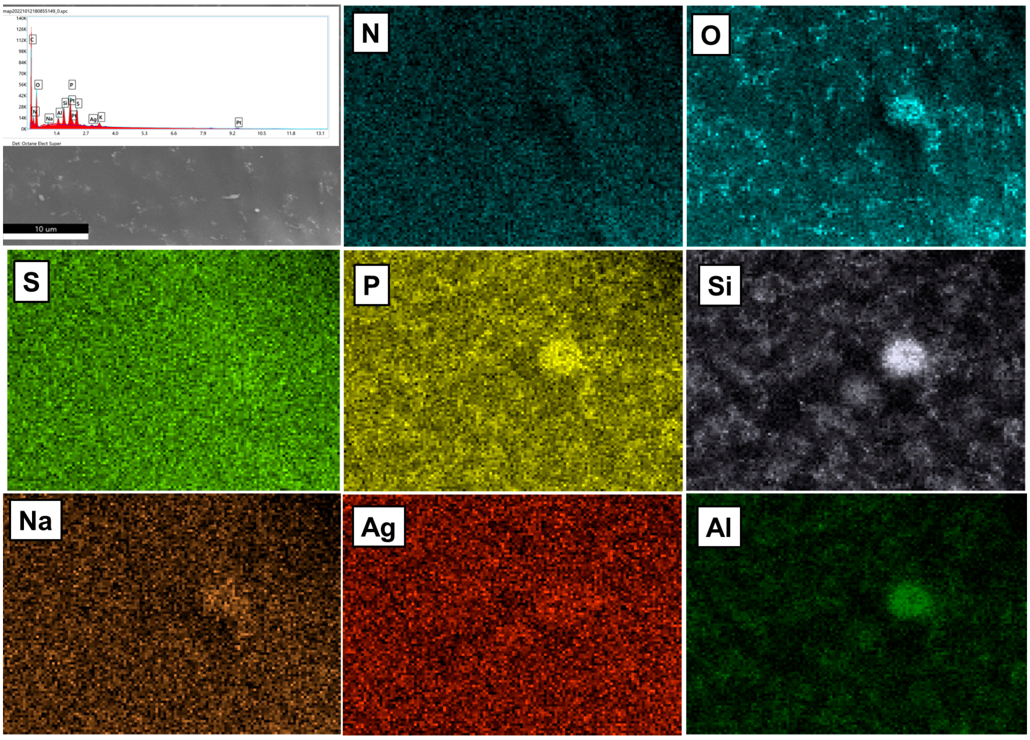

Figure S6. EDX mapping of EPZ-3 residue.
